# A new vision of *Panax ginseng* leaf polysaccharide function: multiple roles in improving growth, flesh quality and muscle energy metabolism of sub-adult grass carp (*Ctenopharyngodon idella*)

**DOI:** 10.1186/s40104-025-01256-z

**Published:** 2025-11-14

**Authors:** Jianrong Yang, Pei Wu, Weidan Jiang, Yang Liu, Yaobin Ma, Xiaowan Jin, Hongmei Ren, Hequn Shi, Xiaoqiu Zhou, Lin Feng

**Affiliations:** 1 Animal Nutrition Institute, Sichuan Agricultural University, Chengdu, Sichuan 611130 China; 2 Fish Nutrition and Safety Production University Key Laboratory of Sichuan Province, Sichuan Agricultural University, Chengdu, 611130 China; 3 Key Laboratory of Animal Disease-Resistance Nutrition, Ministry of Education, Ministry of Agriculture and Rural Affairs, Key Laboratory of Sichuan Province Chengdu, Sichuan, 611130 China; 4 Guangzhou Cohoo Biotech Co., Ltd., Guangzhou, 510663 China

**Keywords:** Energy metabolism, Flesh quality, Grass carp (*Ctenopharyngodon idella*), Myofiber, *Panax ginseng* leaf polysaccharide

## Abstract

**Background:**

As living standards improve, consumers are placing greater emphasis on the enhancement of fish flesh quality, making its improvement increasingly critical. Plant-derived polysaccharides positively affect the improvement of animal flesh quality. *Panax ginseng* leaf polysaccharides (PGLP) have a similar composition and lower cost compared with *Panax ginseng* root polysaccharides. However, its function and application effects in grass carp (*Ctenopharyngodon idella*) are unclear.

**Methods:**

A total of 540 sub-adult grass carp (679 ± 1.29 g), one of the important economic fish species, were used as experimental models and fed diets supplemented with 0, 100, 200, 300, 400, or 500 mg/kg PGLP for 60 d. After 60 d, grass carp were weighed, and their muscles were collected to explore the effects of PGLP on the growth and development of myofibers and energy metabolism-related parameters.

**Results:**

Our study found that PGLP increased the growth performance and muscle nutritional composition as well as improved muscle hardness, springiness, cohesiveness, chewiness, and hyperplasia of myofibers of sub-adult grass carp. Besides, PGLP promoted muscle energy metabolism by increasing creatine, glycogen, pyruvate, and acetyl-CoA contents and creatine kinase (CK), pyruvate kinase (PK), phosphofructokinase (PFK), and hexokinase (HK) activities, while decreasing lactate dehydrogenase (LDH) activity and lactate content in fish muscle. Finally, our study found that PGLP enhanced mitochondrial function by increasing the protein expression of mitochondrial complexes I–V, biogenesis, and fusion and decreasing autophagy and fission in fish muscle.

**Conclusions:**

PGLP improved growth performance and flesh quality of sub-adult grass carp, which may be related to enhancing hyperplasia of myofibers by promoting energy metabolism. We concluded that the recommended amount of PGLP in sub-adult grass carp feed to optimize growth performance is 100–200 mg/kg. This study provides a theoretical basis for the application of PGLP in fish feed and for the analysis of the mechanism of nutrition and feed regulating fish flesh quality, which is of great significance.

**Graphical Abstract:**

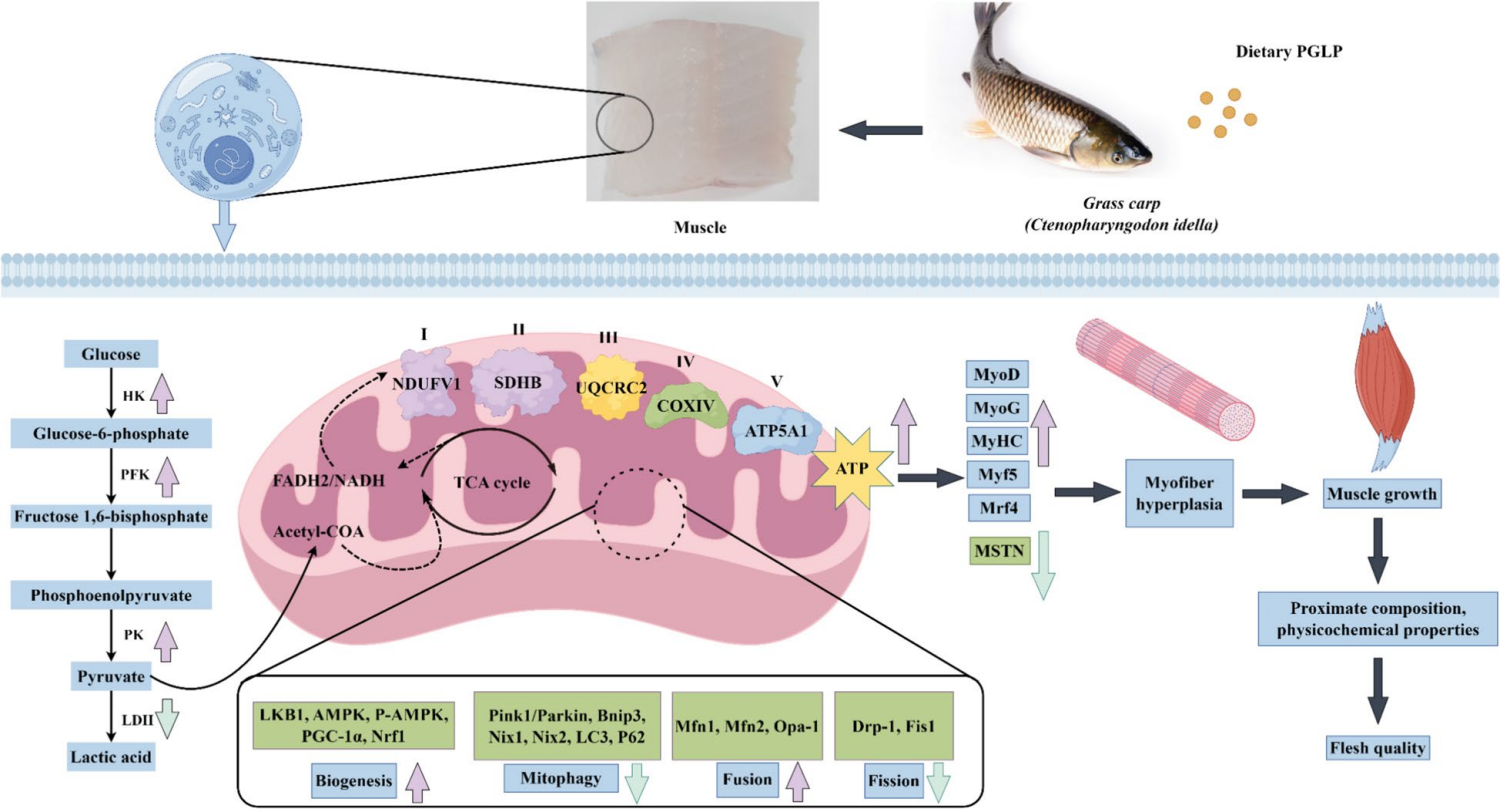

**Supplementary Information:**

The online version contains supplementary material available at 10.1186/s40104-025-01256-z.

## Introduction

*Panax ginseng* (*Panax ginseng* C. A. Meyer) polysaccharides are extracts from a traditional Chinese herbal medicine, known for various functions, including antioxidant properties [1] and promotion of energy metabolism [2]. The content of common *Panax ginseng* root polysaccharides (PGRP) is high, but the extraction cost is high and the harvest period is long (4–6 years). In contrast, *Panax ginseng* leaf polysaccharides (PGLP) have a lower polysaccharide content but are more cost-effective, with a shorter annual harvest period. Meanwhile, both PGRP and PGLP primarily contain pectin, rich in type I rhamnogalacturonic acid (RG-I) and high galacturonic acid (HG) [3, 4]. Therefore, PGLP may exhibit functions similar to those of PGRP, such as regulating energy metabolism and promoting animal growth. Muscle, which constitutes approximately 50% of the body weight in fish, is the primary edible portion. As living standards improve, consumers are placing greater emphasis on the enhancement of flesh quality, making its improvement increasingly critical. Therefore, it is of great significance to develop effective and healthy strategies such as feed technology to improve fish quality. Studies have shown that astragalus (*Radix Astragali*) polysaccharides (APS) [5] and Chinese yam (*Dioscorea *spp.) polysaccharides [6] respectively improved the immunity of grass carp and the richness of intestinal microorganisms, as well as their resistance to *Aeromonas hydrophila*. Meanwhile, soluble soybean (*Glycine max* (L.) Merr.) polysaccharides [7] and tamarind seed polysaccharides (*Tamarindus indica*) [8] inhibited the protein denaturation, water holding capacity decline, and physical instability of grass carp surimi caused by frozen storage, respectively. Besides, it has been found that APS improved the muscle texture properties of the Furong crucian carp (Furong carp♀ × red crucian carp♂) [9]. *Ulva lactuca* polysaccharides alleviated the decrease of hardness, springiness and chewiness of bass (*Lateolabrax maculatus*) fillets induced by cold storage [10]. In summary, PGLP may promote fish flesh quality. It not only provides a new feed technology to improve flesh quality, but also provides a theoretical basis for the application of PGLP in aquatic feed. However, the effect of PGLP on flesh quality has not been investigated, and further research is needed.

Flesh quality is affected by skeletal muscle growth and development, which are regulated by myogenic transcription factors. Myogenic regulatory factors (MRFs) play a vital role in the hyperplasia and hypertrophy of myofibers [11, 12]. In addition, myostatin (Mstn) inhibits the proliferation and differentiation of myofibers [13]. It has been demonstrated that APS increased the diameter and density of myofibers in C2C12 cells after TNF-α stimulation [14]. Until now, there has been no reports published on the effect of PGLP on the growth and development of grass carp myofibers. Therefore, conducting relevant research is essential.

The growth and development of myofibers positively influence flesh quality, a process closely linked to an adequate energy supply. In muscle, adenosine triphosphate (ATP) is mainly produced by three pathways: the phosphate system, oxidative phosphorylation (OXPHOS), and glycolysis, which together maintain the balance of energy metabolism [15]. It has been demonstrated that PGRP increased ATP content and creatine kinase (CK) activity in hepatocytes of chronic hypoxia model mice [2]. Moreover, *Panax ginseng* acidic polysaccharides from the root enhanced the content of glycogen and CK activity in muscle of fatigue model mice [16]. Although the composition of PGLP is similar to that of PGRP, its effect on energy metabolism has not been reported and requires further investigation.

Mitochondria are the primary sites of ATP production, and the stability of their morphology and quality is crucial for mitochondrial structure and function [17]. Previous studies have found that alterations in mitochondrial morphology, such as elongation, swelling, and cristae loss, in the hippocampus of mice exposed to chronic aluminum, result in a decrease in mitochondrial ATP content [18]. Mitochondrial morphology is regulated by mitochondrial dynamics, while their quantity and quality are determined by mitochondrial biogenesis and autophagy. As an important signaling pathway, the liver kinase B1 (LKB1)/activated AMP-activated protein kinase (AMPK)/proliferator-activated receptor gamma coactivator-1α (PGC-1α)/nuclear respiratory factor 1 (NRF1) pathway promotes mitochondrial biogenesis in cultured rat cortical neurons [19]. The PTEN-induced putative kinase 1 (PINK1)/E3 ubiquitin-protein ligase parkin (Parkin) and BCL2-interacting protein 3 (Bnip3)/Bnip3L (NIX) pathways are involved in mitophagy [20, 21]. When the mRNA expression levels of mitofusin 1/2 (*mfn1/2*) and optic atrophy-1 (*opa-1*) are reduced, mitochondrial fusion defects occur [22]. Dynamin-related protein-1 (DRP-1) and fission 1 homolog protein (FIS1) play indispensable roles in mitochondrial fission [23]. However, the effects of PGLP on mitochondrial biogenesis, autophagy, fusion, and fission have not been reported. A previous study found that PGRP alleviated the swelling, cristae breakage, and vacuolization of muscle mitochondria in mice caused by forced swimming [24]. It has been shown that PGRP reduced the mitochondrial swelling in mouse liver cells induced by Fe^2+^-L-Cys [2]. *Panax ginseng* acidic polysaccharides from the root enhanced the protein expression of PGC-1α in muscle of mice [16]. Therefore, PGLP may improve the morphology and structure of mitochondria, providing a foundation for future research.

Until now, it remains unclear whether PGLP can promote the growth and development of myofibers by enhancing energy metabolism. Grass carp, one of the important economic fish species, is highly favored by consumers. Therefore, we used sub-adult grass carp as the experimental subjects to observe the regulatory effect and mechanism of PGLP on flesh quality from the perspective of muscle energy metabolism and the growth and development of myofibers. Meanwhile, the optimal addition amount of PGLP in grass carp feed was determined, providing a theoretical basis for its application in fish feed.

## Materials and methods

### Experimental design and diet

The experimental diet formula and nutrients are shown in Table S1. The added form of PGLP is *Panax ginseng* leaf polysaccharide powder, which contains ≥ 20% PGLP and is provided by Guangzhou Cohoo Biotech Co., Ltd. Large pellet feed ingredients were ground using a universal impact mill (ZY-N04, Jiangsu, China). In this experiment, we weighed PGLP and microcrystalline cellulose according to the design level of PGLP and mixed them evenly to form PGLP premix to ensure more uniform mixing of the feed. Subsequently, the basal diet was first mixed and divided into six portions on average, and then different levels of PGLP premix were added to be fully mixed to form 6 levels of 0, 100, 200, 300, 400, and 500 mg/kg PGLP diet. After all components were completely mixed, the powder was first mixed with water, and the powder was pelletized by the ring mold granulator (HJK-25) and then sent to the hot air circulation oven (CT-C-III) for drying.

### Experimental fish and feeding management

Healthy grass carp (500–600 g) were purchased from a farm (Deyang, Sichuan Province, China) and transported to the test site (Ya'an, Sichuan Province, China) by a fish transport vehicle. After 2 weeks of acclimation period, the fish were selected after adapted temporarily to the cage culture and artificial feeding mode. A total of 540 healthy sub-adult grass carp (679 ± 1.29 g) were selected and randomly assigned to 18 cages (2 m × 2 m × 1.5 m), each treatment 3 cages, and each cage 30 fish. The experiment lasted for 60 d. Saturation feeding was carried out at 7:00, 11:00, 15:00, and 19:00 every day. After every 20 min, the feed intake was observed, and the remaining substances were accurately recorded in each cage. Water treatment was carried out after every 2 d, and regular disinfection was carried out regularly. The professional multiparameter instrument (YSI Incorporated, Yel-low Springs, OH, USA) was used to measure water quality. Under outdoor breeding conditions, the water temperature, pH value, dissolved oxygen (DO), and nitrite were maintained at 27 ± 2 °C, 7.5 ± 0.5, ≥ 6.0 mg/L, and 0.10 ± 0.05 mg/L, respectively.

### Sample collection and assessment

At the end of the experiment, grass carp were weighed to calculate the growth performance according to Table S2. Once the experiment was completed, 12 fish were randomly collected from each treatment, anesthetized with tricaine methanesulfonate (MS-222), and then killed. After the fish were sacrificed, the muscle tissue was quickly dissected and isolated. The left dorsal muscle was divided into three parts: a portion was used to determine pH, a portion was used to determine cooking loss and texture characteristics, and the rest was rapidly frozen in liquid nitrogen and then stored at −80 °C for real-time fluorescence quantitative PCR, Western blotting, and biochemical indicator detection. Meanwhile, three portions of muscle tissues were collected from the right dorsal muscle of grass carp for the determination of muscle nutrients and morphological analysis, respectively [25].

Muscle nutrients (moisture, crude protein, crude lipid) were measured in muscle according to the AOAC [26]. The pH value was measured at 0 h and 24 h after slaughter of live fish. The muscle of 6 fish in each treatment was taken, and the pH value of the center of the meat sample in each group was measured by a pH meter (H29025 portable pH meter, Hanna, Italy). As claimed by Xiao et al. [27], the cooking loss and flesh rate were determined. The hardness, springiness, cohesiveness, and chewiness of muscle tissue were measured by TPA mode in the texture analyzer (CTX000000, AMETEK BOLFE) after the determination of the cooking loss.

The contents of ATP, lactic acid, glycogen, pyruvate, creatine, and acetyl-CoA, and the activities of CK, pyruvate kinase (PK), phosphofructokinase (PFK), hexokinase (HK), and lactate dehydrogenase (LDH) in muscle were assessed by kits. The purchase company and kit number of the kit are shown in Table S3.

### Morphological observation

Firstly, the muscle tissues were stored in a 4% paraformaldehyde solution. Meanwhile, the muscle tissues were cut, dewaxed, paraffin-embedded, and stained with hematoxylin and eosin. Finally, the Nikon Optical Microscope (TS100) was used to observe and used ImageJ (version 1.54f, National Institutes of Health, Bethesda, MD, USA) to count the diameter and density of myofibers of six treatments, each with three replicates.

Moreover, transmission electron microscopy was employed to observe the parameters of myofibrils. Firstly, the muscle tissues were stored in a 2.5% glutaraldehyde solution. Secondly, the muscle tissues were fixed in 1% osmic acid. Then, the muscle tissues were dehydrated in alcohol and acetone. Next, muscle tissues were infiltrated and embedded. Then, the muscle tissues were sectioned and stained in a uranyl acetate saturated alcohol solution (2%) and a lead citrate solution (2.6%). Finally, the sections were observed and images captured using transmission electron microscopy (JEM1400-FLASH). The 0, 100, and 500 mg/kg PGLP groups contained three replicates, respectively.

### Immunofluorescence (IF)

The muscle tissue sections were subjected to antigen repair, blocking, primary antibody incubation (4 °C, over 17 h), secondary antibody incubation (room temperature, 1 h), DAPI staining (10 min, C1006, Beyotime, China), and mounting [28]. Finally, images were captured using an inverted fluorescence microscope (Leica DMI4000B, Germany) and ImageJ (version 1.54f, National Institutes of Health). Details of the proteins are as follows: autophagy receptor P62 (P62, HA721171, 1:600) and microtubule-associated protein 1 light chain 3 (LC3, ET1701-65, 1:100) are derived from HUABIO (Hangzhou, Zhejiang, China).

### Real-time quantitative PCR

RNA was obtained from muscle tissues, and cDNA was obtained by reverse transcription [29]. According to the primer sequence in Table S4, the expression level of the target gene was quantified by 2^−ΔΔCT^ with the expression level of β-actin as the standard.

### Western blot analysis

The method was used, as claimed by Xiao et al. [27], to extract total muscle proteins and determine protein concentration. Firstly, the target protein was transferred to a PVDF membrane by SDS-PAGE and wet transfer. Next, followed by closed primary antibody combination and secondary antibody combination. Finally, imaging and protein quantification were performed using ECL kits (Beyotime Biotechnology Co., Ltd., China) and ImageJ (version 1.54f, National Institutes of Health) software, respectively. Details of the proteins are as follows: NADH-ubiquinone oxidoreductase core subunit V1 (NDUFV1, 11238-1-AP, 1:5,000), succinate dehydrogenase B (SDHB, ER1803-63, 1:1,000), ubiquinol-cytochrome c reductase core protein 2 (UQCRC2, HA721872, 1:2,000), cytochrome c oxidase (COX IV, ET1701-63, 1:3,000), ATP synthase-α (ATP5A1, ET1703-53, 1:1,000), p-AMPK (ET1701-37, 1:1,000), LKB1 (HA500143, 1:800), Nrf1 (ET1705-86, 1:2,000), PGC-1α (ET1702-96, 1:1,000), β-actin (HA722023, 1:20,000) derive from HUABIO (Hangzhou, Zhejiang, China), AMPK (A12718, 1:750) are derived from ABclonal (Wuhan, Hubei, China).

### Statistical analysis

SPSS 22.0 software (SPSS Inc., Chicago, IL, USA) was used to analyze the data by one-way analysis of variance (ANOVA) and Duncan's multiple interval test to determine the difference. When *P* < 0.05, the difference was statistically significant. Furthermore, the linear and quadratic effects of varying doses of PGLP were analyzed by Orthogonal polynomial contrasts. Data were expressed as mean ± standard deviation (SD).

## Result

### PGLP affected growth performance, approximate composition and texture of muscle in sub-adult grass carp

PGLP had trends to increase FBW, WG, PWG, SGR, FI, and FE of sub-adult grass carp at 100 and 200 mg/kg (*P* < 0.1) (Table 1). FCR showed the opposite trend, which had trend to reduce at 100 and 200 mg/kg PGLP (*P* < 0.1). The CF remarkably decreased with the increase of PGLP level and reached the minimum at 200 mg/kg (*P* < 0.05). Besides, flesh rate was remarkably enhanced at 100–500 mg/kg PGLP (*P* < 0.05).

**Table 1:** Effects of PGLP on growth performance of sub-adult grass carp

Item	Dietary PGLP levels, mg/kg diet						*P*-values	
	0	100	200	300	400	500	Linear	Quadratic
IBW, g/fish	680.11±1.50	679.44±0.96	678.33±0.00	679.44±1.92	678.89±0.96	679.44±1.92	0.055	0.803
FBW, g/fish	1,661.33±24.17^A^	1,735.89±37.07^B^	1,733.11±26.53^B^	1,724.67±56.22^AB^	1,660.22±61.55^A^	1,699.89±32.69^AB^	0.838	0.084
WG, g/fish	981.22±22.83^A^	1,056.44±37.24^B^	1,054.78±26.53^B^	1,045.22±55.67^AB^	981.33±62.18^A^	1,020.44±34.61^AB^	0.853	0.080
PWG, %	144.27±3.08^A^	155.49±5.53^B^	155.50±3.91^B^	153.83±8.10^AB^	144.56±9.30^A^	150.20±5.51^AB^	0.877	0.075
SGR, %/d	1.49±0.02^A^	1.56±0.04^B^	1.56±0.03^B^	1.55±0.05^AB^	1.49±0.06^A^	1.53±0.04^AB^	1.000	0.087
FI, g/fish	1,484.56±10.01^A^	1,514.22±14.79^B^	1,512.83±10.61^B^	1,509.73±22.64^AB^	1,483.81±24.46^A^	1,499.82±12.60^AB^	0.828	0.086
FE	0.66±0.01^A^	0.70±0.02^B^	0.70±0.01^B^	0.69±0.03^AB^	0.66±0.03^A^	0.68±0.02^AB^	0.818	0.106
FCR	1.51±0.03^BC^	1.43±0.04^A^	1.44±0.03^A^	1.45±0.06^AB^	1.52±0.07^C^	1.47±0.04^ABC^	0.831	0.081
CF, g/cm3	1.95±0.13^bc^	1.83±0.11^b^	1.55±0.05^a^	1.92±0.14^bc^	1.96±0.09^c^	1.98±0.03^c^	0.011	<0.001
Flesh rate, %	64.31±0.82^a^	68.01±3.24^bc^	69.64±1.89^c^	67.41±0.52^bc^	67.34±1.37^bc^	66.73±2.47^b^	0.076	<0.001

*IBW* Initial body weight, *FBW* Final body weight, *WG* Weight gain, *PWG* Percent weight gain, *SGR* Specific growth rate, *FI* Feed intake, *FE* Feed efficiency, *FCR* Feed conversion rate, *CF* Condition factor

Data are presented as mean ± SD (*n* = 3; *n* = 6 for CF and Flesh rate. The Plinear and Pquadratic indicate the significance of the linear and quadratic dose-response relationships, respectively

^A-C^Mean values within a row with different superscript letters indicate significant trends (0.05 ≤ *P* < 0.1)

^a-c^Mean values within a row with different superscript letters indicate significant different (P < 0.05)

As shown in Table 2, there was no remarkable difference in muscle moisture (*P* > 0.05), and crude protein content was remarkably enhanced at 100–400 mg/kg PGLP (*P* < 0.05), as well as the crude lipid content remarkably declined and reached the minimum at 100 mg/kg (*P* < 0.05). The hardness, springiness, cohesiveness, and chewiness of muscle were remarkably increased at 200–500, 100–500, 200–300, and 300 mg/kg PGLP, respectively (*P* < 0.05) (Table 3). The muscle pH_0h_ was remarkably declined at 200 and 300 mg/kg PGLP (*P* < 0.05). The muscle pH_24h_ had the same trend as pH_0h_, but it was not a remarkable difference (*P* > 0.05). Besides, the cooking loss of muscle was remarkably declined at 100–300 mg/kg PGLP (*P* < 0.05).

**Table 2:** Effects of PGLP on muscle proximate composition of sub-adult grass carp

Item	Dietary PGLP levels, mg/kg diet						*P*-values	
	0	100	200	300	400	500	Linear	Quadratic
Moisture, %	76.39 ± 0.58^ab^	75.95 ± 0.36^a^	76.95 ± 0.61^b^	76.96 ± 1.05^b^	76.99 ± 0.51^b^	77.08 ± 0.56^b^	0.006	0.622
Crude lipid, %	1.83 ± 0.05^b^	1.67 ± 0.07^a^	1.70 ± 0.08^a^	1.74 ± 0.05^a^	1.87 ± 0.05^b^	2.19 ± 0.09^c^	< 0.001	< 0.001
Crude protein, %	20.06 ± 0.06^a^	21.09 ± 0.03^e^	20.28 ± 0.03^d^	20.19 ± 0.09^c^	20.15 ± 0.04^bc^	20.11 ± 0.04^ab^	< 0.001	< 0.001

Data are presented as mean ± SD (*n* = 6). ^a^^–d^Mean values within a row with different superscript letters are significantly different (*P* < 0.05). The *P*_*linear*_ and *P*_*quadratic*_ indicate the significance of the linear and quadratic dose-response relationships, respectively

**Table 3:** Effects of PGLP on muscle structure characteristics, water holding capacity and pH value of sub-adult grass carp

Item	Dietary PGLP levels, mg/kg diet						*P*-values	
	0	100	200	300	400	500	Linear	Quadratic
Hardness, g	150.17 ± 16.83^a^	153.97 ± 10.42^ab^	227.30 ± 20.04^c^	287.22 ± 32.02^d^	176.05 ± 12.79^b^	174.07 ± 14.26^b^	0.001	< 0.001
Springiness, mm	9.92 ± 0.02^a^	9.94 ± 0.01^b^	9.96 ± 0.00^c^	9.95 ± 0.00^bc^	9.95 ± 0.02^bc^	9.95 ± 0.00^bc^	< 0.001	< 0.001
Cohesiveness	0.248 ± 0.025^a^	0.278 ± 0.034^ab^	0.282 ± 0.019^b^	0.283 ± 0.029^b^	0.278 ± 0.025^ab^	0.272 ± 0.012^ab^	0.178	0.028
Chewiness, mJ	4.82 ± 0.83^b^	4.92 ± 0.61^b^	5.28 ± 0.52^b^	7.33 ± 0.67^c^	4.63 ± 0.50^b^	3.65 ± 0.38^a^	0.030	< 0.001
pH_0h_	6.61 ± 0.06^c^	6.53 ± 0.11^bc^	6.35 ± 0.05^a^	6.46 ± 0.04^b^	6.55 ± 0.04^c^	6.60 ± 0.08^c^	0.556	< 0.001
pH_24h_	6.08 ± 0.01	6.06 ± 0.01	6.06 ± 0.04	6.08 ± 0.04	6.09 ± 0.02	6.09 ± 0.04	0.158	0.230
Cooking loss, %	15.37 ± 0.98^c^	13.84 ± 0.71^b^	13.78 ± 0.81^b^	11.83 ± 0.86^a^	14.58 ± 0.70^bc^	15.19 ± 0.80^c^	0.820	< 0.001

Data are presented as mean ± SD (*n* = 6). ^a^^–^^d^Mean values within a row with different superscript letters are significantly different (*P* < 0.05). The *P*_*linear*_ and *P*_*quadratic*_ indicate the significance of the linear and quadratic dose-response relationships, respectively

### PGLP affected the growth and development of myofibers in sub-adult grass carp

**Fig. 1:**
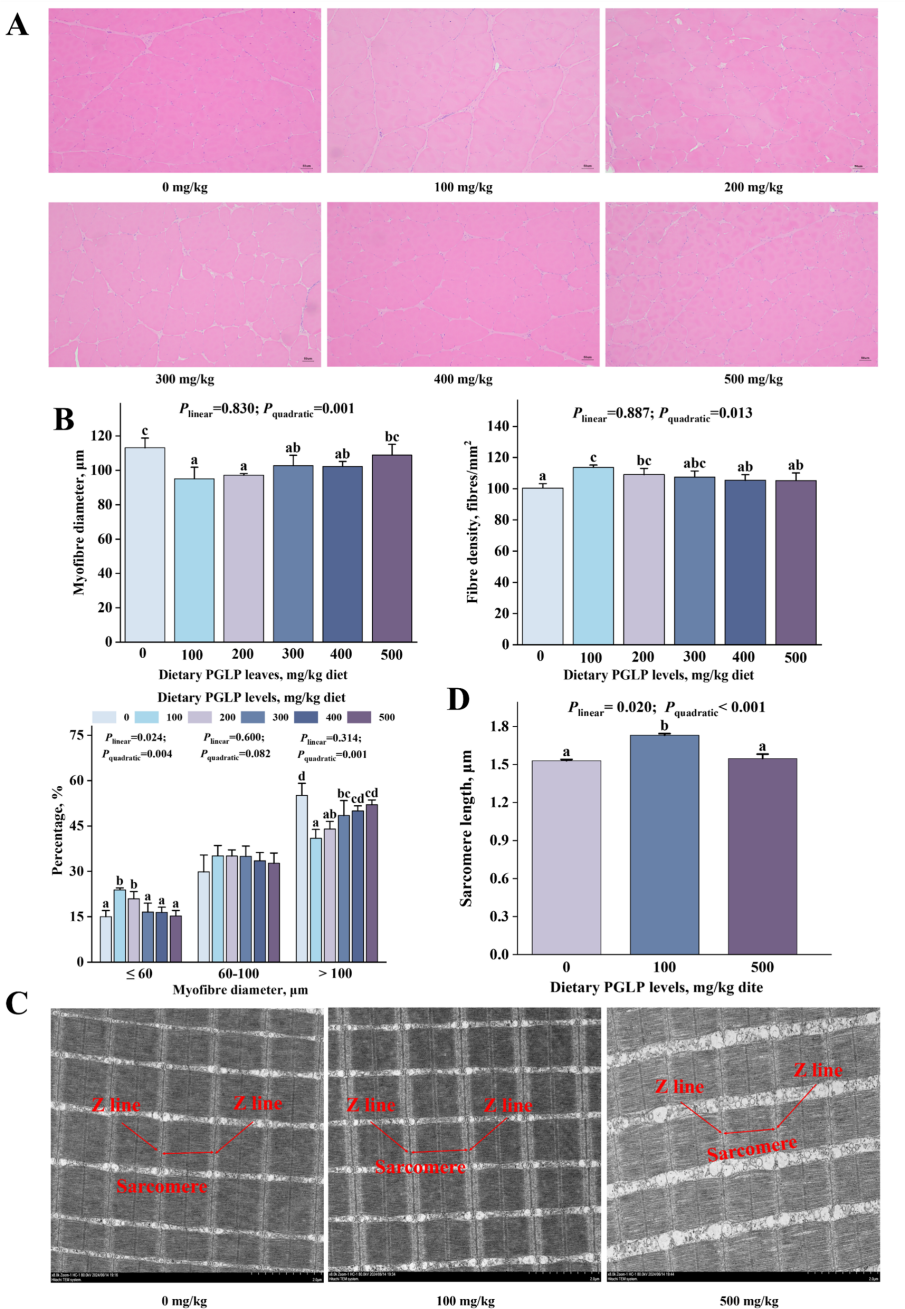
Effects of PGLP on growth and development of myofibers of grass carp. **A** Cross-sectional microstructure of grass carp muscle (× 100, scale bar = 50 μm). **B** Myofibers diameter, density, and frequency distribution. **C** Transmission electron microscopy imaging of myofibrils (× 8,000, scale bar = 2 μm) of grass carp muscle. **D** sarcomere length. Data are presented as mean ± SD, and the error bars indicate SD (*n* = 3). The *P*_*linear*_ and *P*_*quadratic*_ indicate the significance of the linear and quadratic dose-response relationships, respectively. ^a^^–^^d^Different letters indicate significant differences (*P* < 0.05)

**Fig. 2:**
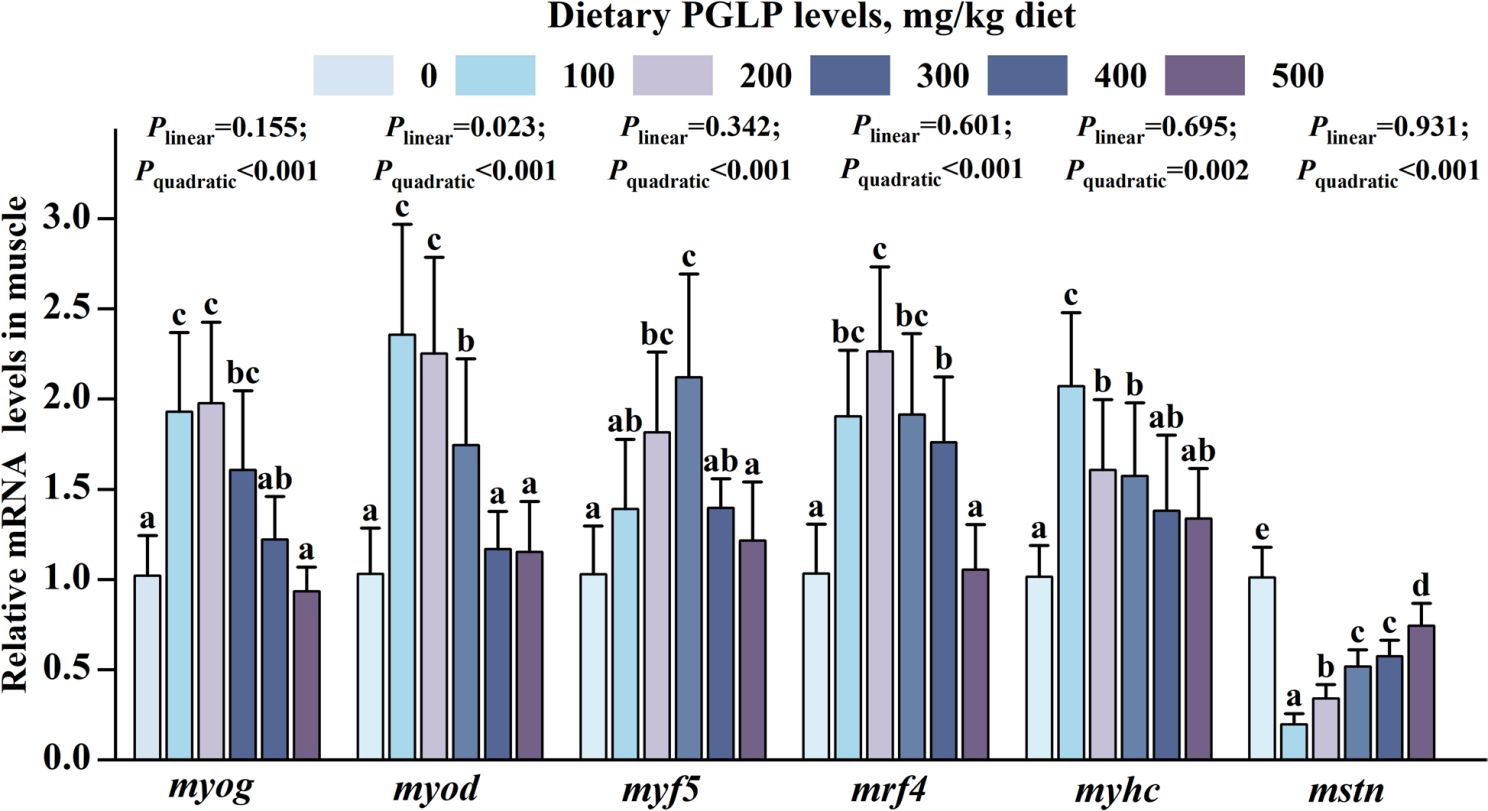
Effects of PGLP on mRNA expression of myogenic regulatory factors in grass carp muscle. Myogenin (*myog*); Myogenic determining factor (*myod*); Myogenic regulatory factor 5 (*myf5*); Myogenic regulatory factors 4 (*mrf4*); Myosin heavy chain (*myhc*). Data are presented as mean ± SD (*n* = 6), error bars indicate SD. The *P*_*linear*_ and *P*_*quadratic*_ indicate the significance of the linear and quadratic dose-response relationship, respectively. ^a^^–^^e^Different letters indicate the significant difference among treatments (*P* < 0.05)

The density of myofibers and frequency of diameter ≤ 60 μm were enhanced remarkably, reaching the maximum at 100 mg/kg (*P* < 0.05) (Fig. 1A and B). Oppositely, the diameter of myofibers and the frequency of diameter > 100 μm declined remarkably and reached the minimum at 100 mg/kg PGLP (*P* < 0.05). Meanwhile, the frequency of myofibers diameter of 60–100 μm was not a significant difference (*P* > 0.05). Besides, PGLP remarkably enhanced the sarcomere length at 100 mg/kg (*P* < 0.05) (Fig. 1C and D). The mRNA expression levels of *myog*, *myod*, *myf5*, *mrf4*, and *myhc* in muscle were remarkably enhanced at 100–300, 100–300, 200–300, 100–400, and 100–300 mg/kg PGLP, respectively (*P* < 0.05) (Fig. 2). Finally, PGLP remarkably declined the mRNA expression level of *mstn* in muscle at 100–500 mg/kg (*P* < 0.05).

### PGLP affected glycolysis of muscle in sub-adult grass carp

**Fig. 3:**
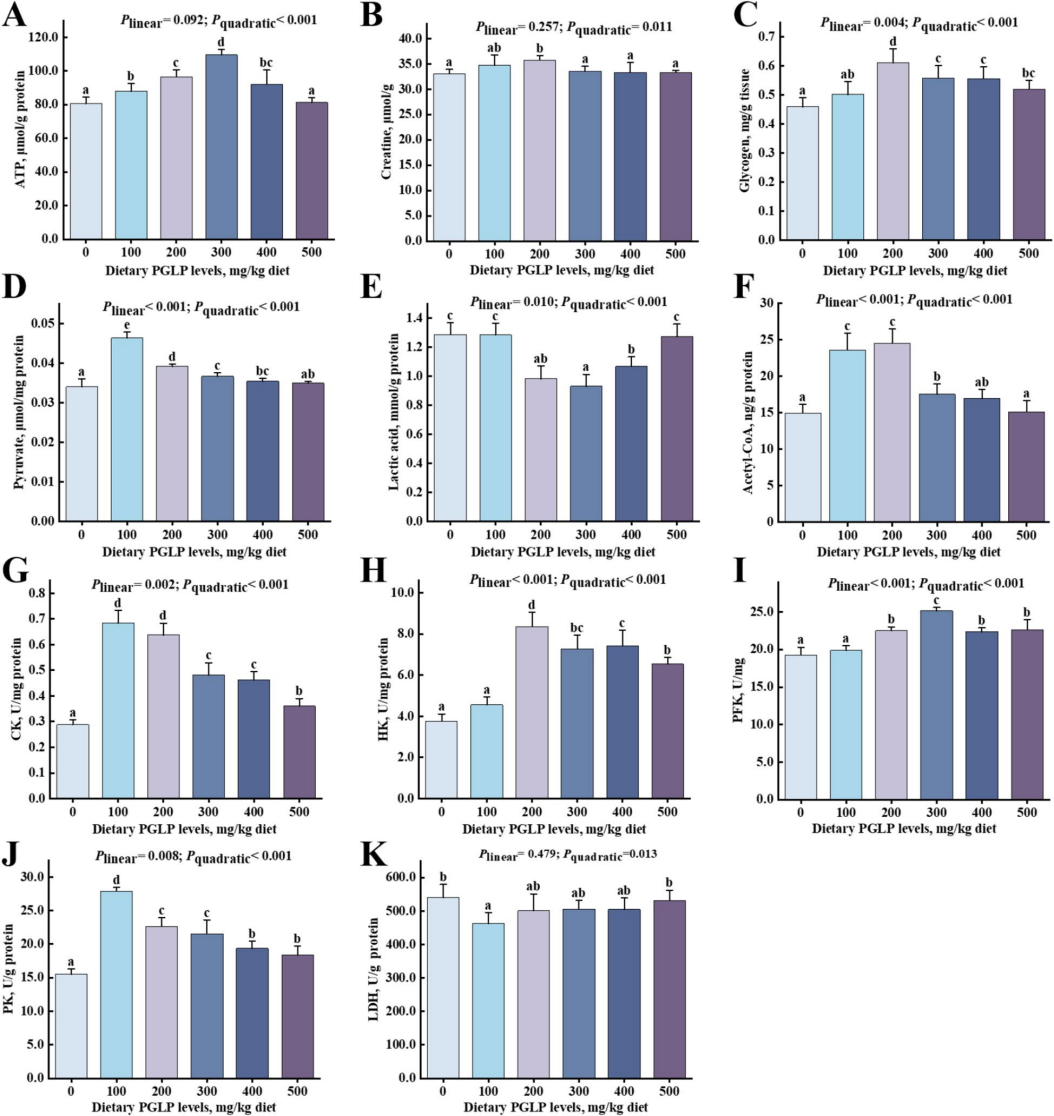
Effects of PGLP on phosphagen and glycolysis metabolism in grass carp muscle. **A** ATP, μmol/g protein. **B** Creatine, μmol/g. **C** Glycogen, mg/g tissue. **D** Pyruvate, μmol/g protein. **E** Lactate, mmol/g protein. **F** Acetyl-CoA, ng/g protein. **G** CK, creatine kinase, U/mg protein. **H** HK, hexokinase, U/mg protein. **I** PFK, phosphofructokinase, U/mg. **J** PK, pyruvate kinase, U/g protein. **K** LDH, lactate dehydrogenase, U/g protein. Data are presented as mean ± SD (*n* = 6), error bars indicate SD. The *P*_*linear*_ and *P*_*quadratic*_ indicate the significance of the linear and quadratic dose-response relationship, respectively. ^a^^–^^e^Different letters indicate the significant difference among treatments (*P* < 0.05)

Compared with the 0 mg/kg PGLP, the muscle ATP, creatine, and glycogen contents and CK activity were remarkably enhanced at 100–400 mg/kg and reached the maximum at 300, 200, 200 and 100 mg/kg PGLP, respectively (*P* < 0.05) (Fig. 3A–C and G). As the increase of PGLP levels, the muscle pyruvate content and HK, PFK, PK activities were remarkably enhanced and reached the maximum at 100, 200, 300, and 100 mg/kg PGLP, respectively (*P* < 0.05) (Fig. 3D and H–J). Besides, the muscle lactic acid content was remarkably decreased at 200–400 mg/kg PGLP and the LDH activity was remarkably declined at 100 mg/kg (*P* < 0.05) (Fig. 3E and K). Finally, compared with 0 mg/kg PGLP group, the content of acetyl-CoA in muscle changed the most at 200 mg/kg (*P* < 0.05) (Fig. 3F).

**Fig. 4:**
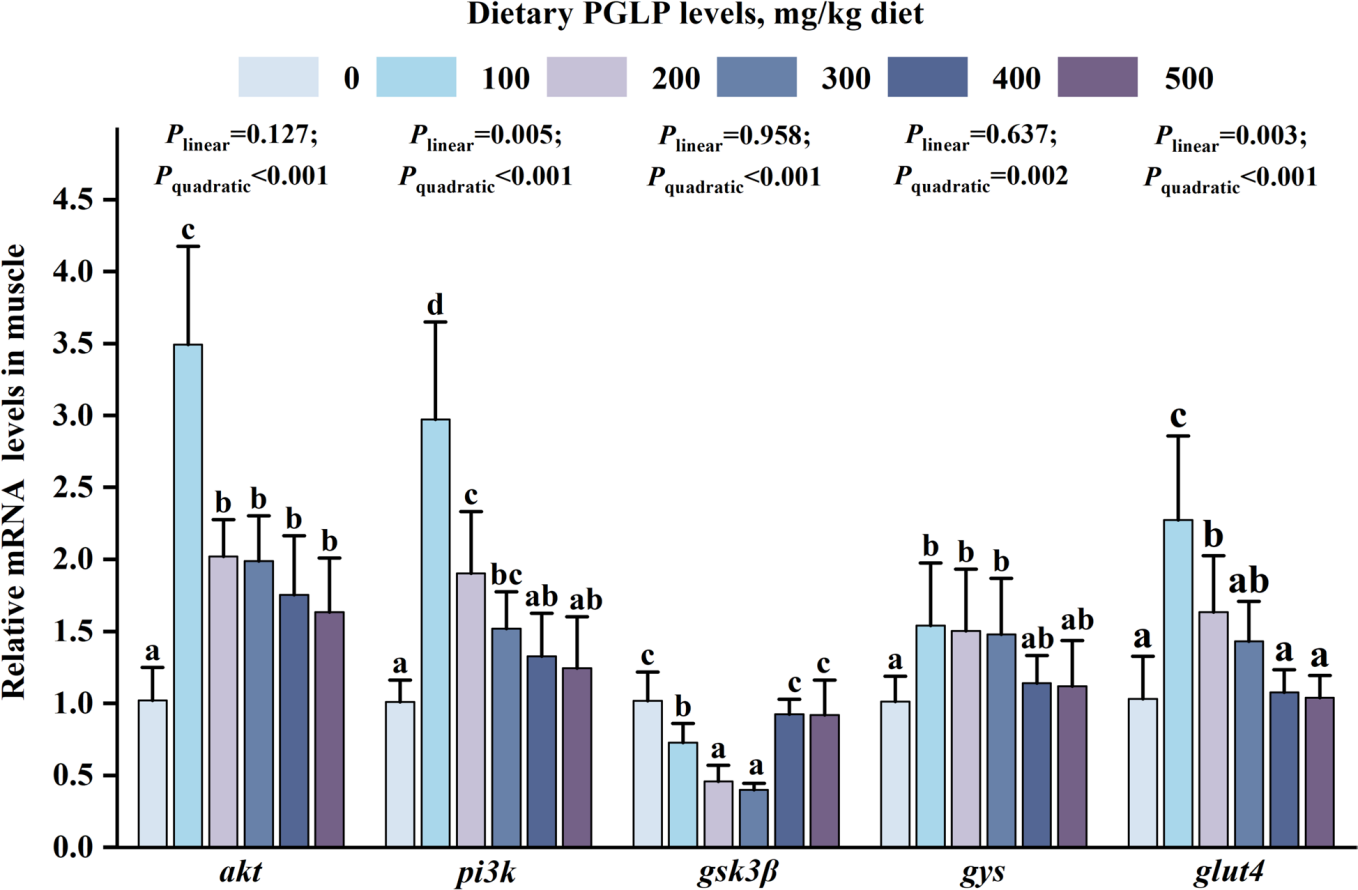
Effects of PGLP on mRNA expression of glycogen synthesis-related genes in muscle of grass carp. Protein kinase B (*akt*); phosphoinositide 3-kinase (*pi3k*); glycogen synthase kinase-3β (*gsk3β*); glycogen synthase (*gys*); glucose transporter-4 (*glut4*). Data are presented as mean ± SD (*n* = 6), error bars indicate SD. The *P*_*linear*_ and *P*_*quadratic*_ indicate the significance of the linear and quadratic dose-response relationships, respectively. Different letters indicate the significant difference among treatments (*P* < 0.05)

It has been found that PGLP significantly enhanced the content of glycogen in muscle (Fig. 3C), so qPCR quantitative analysis of glycogen synthesis-related genes was further carried out. Compared with the 0 mg/kg PGLP, the mRNA expression levels of *akt*, *pi3k*, *gys,* and *glut4* were remarkably up-regulated in muscle and reached the maximum at 100 mg/kg (*P* < 0.05) (Fig. 4). Meanwhile, PGLP remarkably down-regulated the mRNA expression level of *gsk3β* in muscle at 100–300 mg/kg (*P* < 0.05).

### PGLP affected the expression of mitochondrial complex of muscle in sub-adult grass carp

**Fig. 5:**
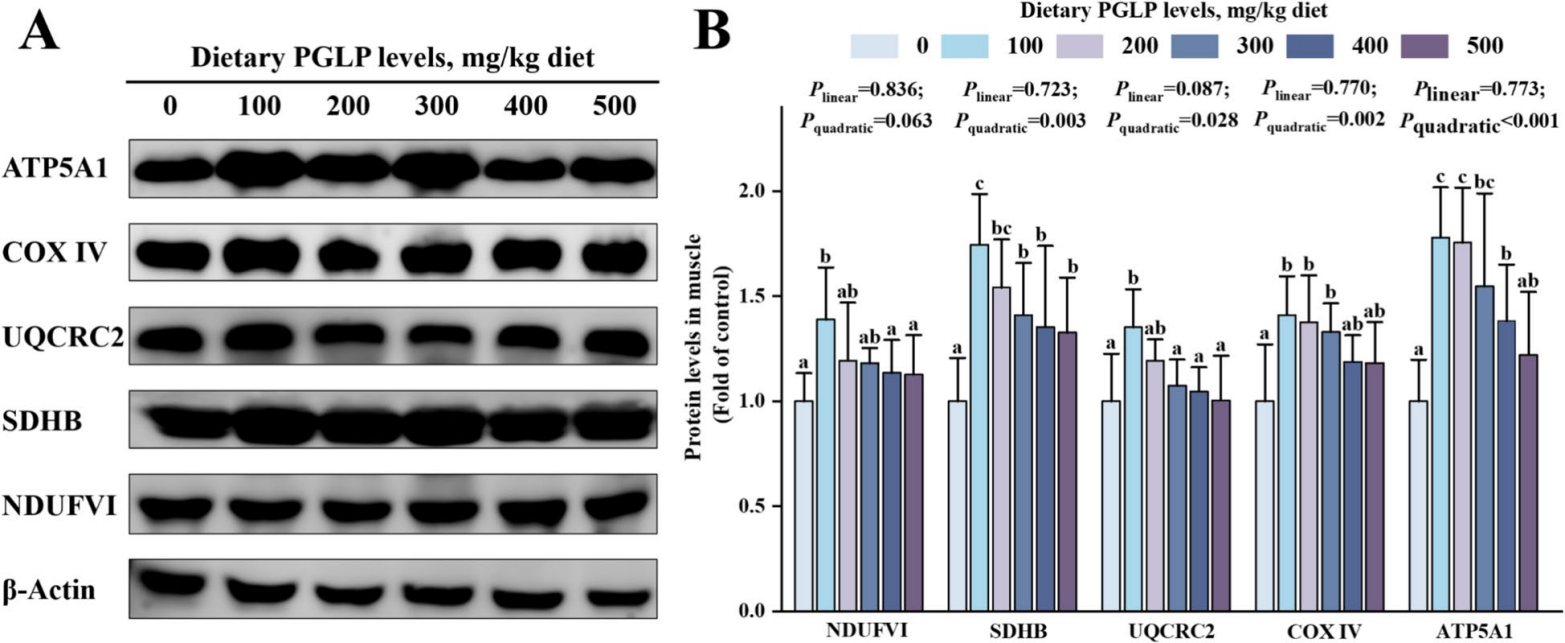
Effects of PGLP on protein expression of mitochondrial respiratory complexes in grass carp muscle. **A** Western blotting bands. **B** Quantification. NDUFV1: NADH-ubiquinone oxidoreductase core subunit V1; SDHB: Succinate dehydrogenase B; UQCRC2: Ubiquinol-cytochrome c reductase core protein 2; COX IV: Cytochrome c oxidase; ATP5A1: ATP synthase-α. Data are presented as mean ± SD (*n* = 6), error bars indicate SD. The *P*_*linear*_ and *P*_*quadratic*_ indicate the significance of the linear and quadratic dose-response relationships, respectively. Different letters indicate the significant difference among treatments (*P* < 0.05)

Our results showed that PGLP remarkably increased the protein expression of NDUFV1 and UQCRC2 in muscle at 100 mg/kg PGLP (*P* < 0.05) (Fig. 5). Meanwhile, the protein expression of SDHB, COX IV, and ATP5A1 showed the same trend and were remarkably enhanced at 100–500, 100–300, and 100–300 mg/kg PGLP, respectively (*P* < 0.05).

### PGLP affected the mitochondrial quality control of muscle in sub-adult grass carp

**Fig. 6:**
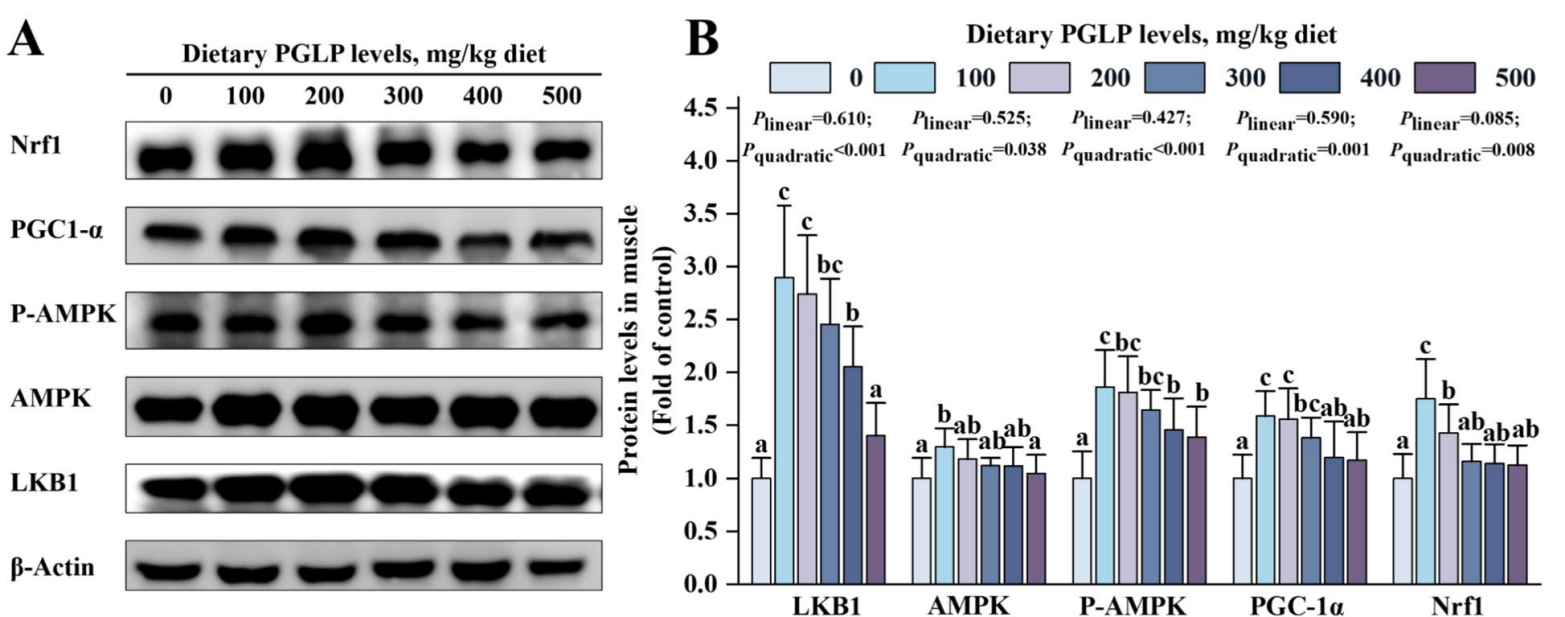
Effects of PGLP on protein expression of biogenesis-related proteins in grass carp muscle. **A** Western blotting bands. **B** Quantification. Data are presented as mean ± SD (*n* = 6), error bars indicate SD. The *P*_*linear*_ and *P*_*quadratic*_ indicate the significance of the linear and quadratic dose-response relationships, respectively. Different letters indicate the significant difference among treatments (*P* < 0.05)

**Fig. 7:**
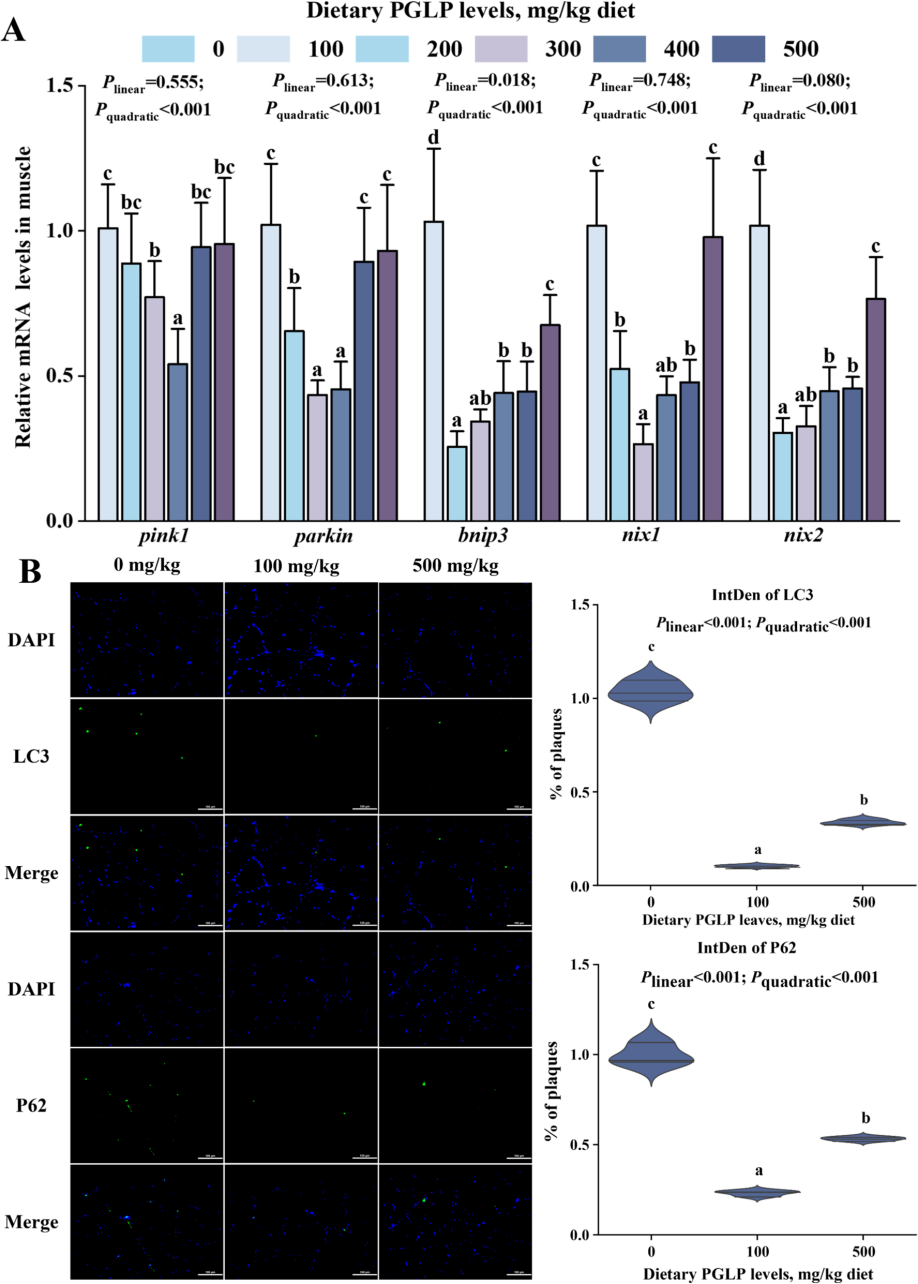
Effects of PGLP on expression of mitophagy-related genes in muscle of grass carp. **A** Effects of PGLP on mRNA expression of *pink1*, *parkin*, *bnip3*, *nix1*, and *nix2* in muscle of grass carp ( *n* = 6). **B** Effects of different PGLP levels on immunofluorescence staining of LC3 and p62 in muscle of grass carp. Scale bar = 100 μm (× 200), *n* = 3 fish. Data are presented as mean ± SD, error bars indicate SD. The *P*_*linear*_ and *P*_*quadratic*_ indicate the significance of the linear and quadratic dose-response relationships, respectively. Different letters indicate the significant difference among treatments (*P* < 0.05)

The protein expressions of LKB1, P-AMPK, PGC-1α, and Nrf1 in muscle were significantly increased and reached the maximum at 100 mg/kg PGLP (*P* < 0.05) (Fig. 6). Meanwhile, PGLP remarkably down-regulated the mRNA expression levels of *pink1*, *parkin*, *bnip3*, *nix1*, and *nix2* in grass carp muscle and reached the minimum at 300, 200, 100, 200 and 100 mg/kg PGLP, respectively (*P* < 0.05) (Fig. 7A). In addition, the protein expression of autophagy marker proteins LC3 and P62 also showed the same trend. Compared with the 0 mg/kg PGLP, the protein expression of LC3 and P62 remarkably decreased in muscle at 100 and 500 mg/kg PGLP (*P* < 0.05) (Fig. 7B).

**Fig. 8:**
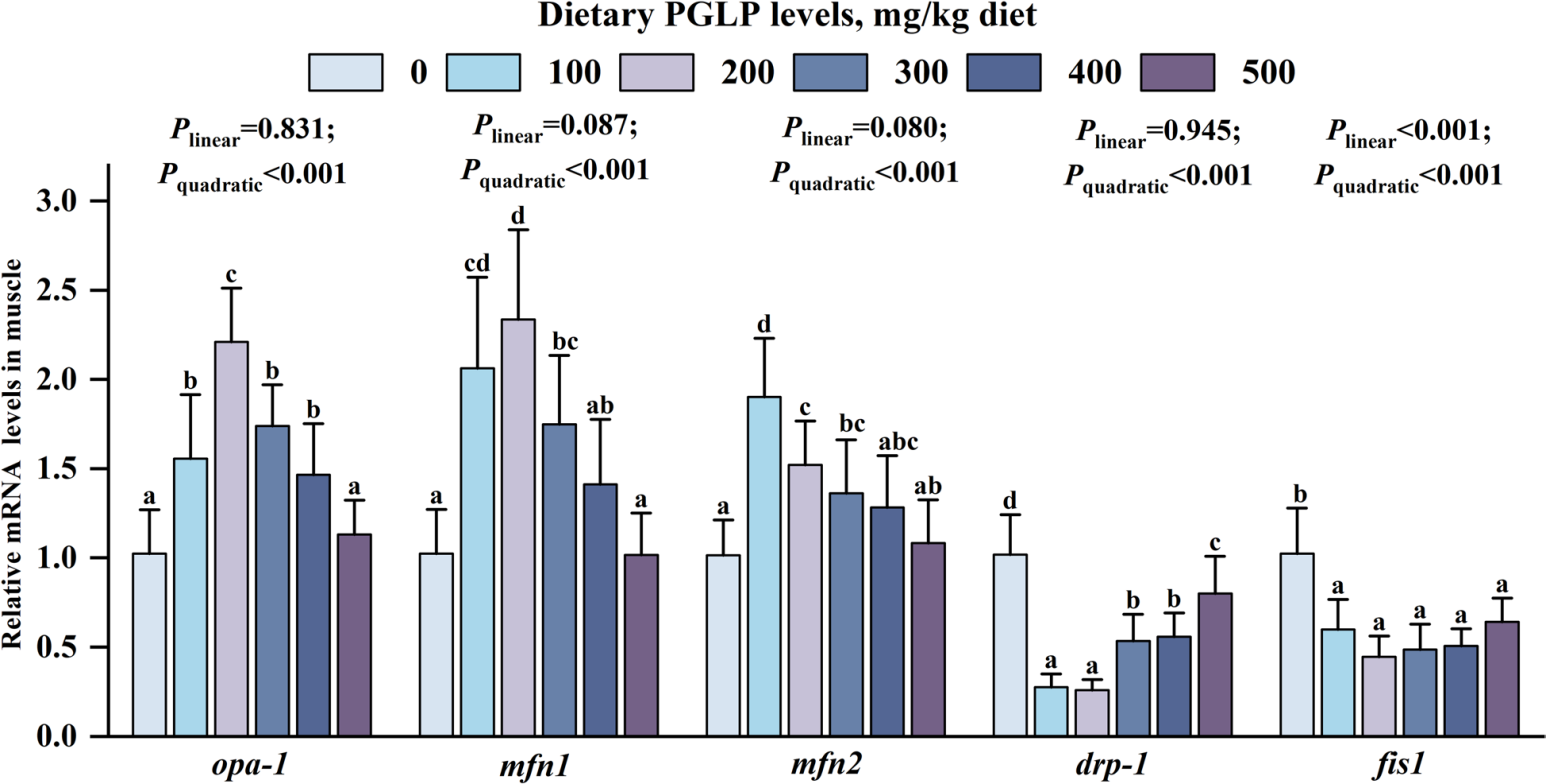
Effects of PGLP on mitochondrial fission and fusion related parameters in muscle of grass carp. Data are presented as mean ± SD (*n* = 6), error bars indicate SD. The *P*_*linear*_ and *P*_*quadratic*_ indicate the significance of the linear and quadratic dose-response relationships, respectively. Different letters indicate the significant difference among treatments (*P* < 0.05)

Our results showed that the mRNA expression levels of *opa-1* and *mfn1/2* in muscle were remarkably enhanced at 100–400 and 100–300 mg/kg PGLP, respectively (*P* < 0.05) (Fig. 8). Meanwhile, PGLP remarkably declined the mRNA expression levels of *drp-1* and *fis1* in muscle at 100–500 mg/kg (*P* < 0.05).

## Discussion

### PGLP promoted growth performance and flesh quality of grass carp

We have studied the effect of PGLP on the growth performance of fish. In our study, PGLP had trends to increase FBW, WG, PWG, SGR, FI, and FE of sub-adult grass carp, indicating that PGLP and PGRP had the same effect on promoting animal growth performance. Previous studies have shown that PGRP promoted the growth of white shrimp [30], weaned piglets [31], and Xuefeng black-bone chickens [32], respectively. Based on our findings, we recommend adding 100–200 mg/kg of PGLP to grass carp feed, as it had trend to improve growth performance. The study found that *Eucommia ulmoides* leaves extract [33] and Mazari palm (*Nannorrhops ritchiana*) leaves extract [34] increased the weight gain of grass carp. In addition, studies on other animals have also proved that plant polysaccharides can promote the production performance of animals. It has been found that dandelion (*Taraxacum mongolicum* Hand.-Mazz.) polysaccharides [35], agaricus blazei (*Agaricus subrufescens*) polysaccharides [36], and APS [37] improved the production performance of laying hens, Korean quails, and weaned piglets, respectively. In summary, improving the application of plant polysaccharides such as PGLP in feed has a positive effect on promoting the growth of animals.

Muscle constitutes about 50% of body weight, and flesh quality significantly influences consumers' purchasing decisions, highlighting the critical need to enhance flesh quality. The flesh quality of fish is determined by nutritional composition, physicochemical properties, and other aspects of muscle [38]. For instance, *Eucommia ulmoides* extract increased the crude protein content of grass carp muscle, and reduced the centrifugal loss, steaming loss, and drip loss_24h_ to improve flesh quality [33]. In our study, PGLP decreased the moisture, crude lipid content, and cooking loss of muscle, alleviated the decline rate of pH, and increased the crude protein content, hardness, springiness, cohesiveness, and chewiness of muscle, demonstrating that PGLP enhanced the flesh quality of fish. The trend of moisture and crude protein was consistent with the results of other plant polysaccharides. For instance, bran polysaccharides increased the crude protein content of carp muscle and reduced moisture and crude ash [39]. The content of crude lipid in muscle is closely related to feed and feeding conditions [40]. Meanwhile, lipid, as an important energy source for animals, can provide the necessary energy for the body's life activities. Therefore, it is speculated that PGLP may reduce the content of crude fat by promoting fat catabolism, but it needs to be further verified. Previous studies have demonstrated that the growth and development of myofibers promoted muscle texture characteristics [41]. Therefore, we next focused on investigating the growth and development of myofibers.

### PGLP enhanced growth and development of myofibers of grass carp

The frequency of myofibers diameter ≤ 60 μm and > 100 μm demonstrates hyperplasia and hypertrophy of myofibers in grass carp, respectively [42]. In this experiment, PGLP enhanced the frequency of diameter ≤ 60 μm and decreased the frequency of diameter > 100 μm, demonstrating that PGLP enhanced hyperplasia of myofibers. The process of growth and development of myofibers are controlled by many regulatory factors and plays a vital role in different processes. Among them, MyoD and Myf5 determine myoblast proliferation, MyoG and Mrf4 maintain differentiation, MyHC is an important marker of proliferation and hypertrophy, and Mstn inhibits myofibers proliferation and differentiation [27]. In this experiment, PGLP promoted the mRNA expression levels of MRFs and inhibited the expression level of *mstn*, indicating that PGLP promoted the hyperplasia of myofibers. It has been claimed that APS increased diameter and density of myofibers in C2C12 cells after TNF-α stimulation [14]. The growth and development of myofibers is inseparable from sufficient energy supply. Therefore, we explored the effect of PGLP on muscle energy metabolism.

### PGLP increased glycolysis and creatine phosphate system

In muscle, ATP is a direct energy source. Li et al. [2] found that PGRP increased ATP content in liver of chronically hypoxic mice. Until now, the effect of PGLP on ATP content in fish is still unclear. In this experiment, PGLP increased the muscle ATP content. In muscle, ATP is mainly derived from the phosphagen system, OXPHOS, and glycolysis [15]. The phosphagen system (CK/PCr system) refers to the reaction in which creatine phosphate and adenosine diphosphate (ADP) are catalyzed by CK to produce creatine and ATP [43]. When the vast majority of creatine phosphate is consumed, the muscle mainly relies on glycolysis to produce ATP [44]. Glycogen is an important substrate for glycolysis [45] and can be decomposed into glucose-1-phosphate, which can enter glycolysis to produce pyruvate under the catalysis of HK, PFK, PK, and other enzymes. Then, pyruvate can be converted to lactate or acetyl-CoA [46]. Our results showed that PGLP increased contents of creatine, glycogen, pyruvate, and acetyl-CoA, as well as CK, HK, PFK, and PK activity, while decreasing LDH activity and lactate content in muscle, indicating that PGLP accelerated the process of the phosphagen system and glycolysis in muscle and reduced anaerobic glycolysis. Until now, the effect of PGLP on muscle glucose metabolism in fish has not been reported. However, it has been found that PGRP enhanced glucose metabolism in the muscle tissue of mice with a fatigue or chronic hypoxia model [2, 16]. Glycogen content increases may be correlated with the glycogen synthesis. PI3K/Akt, GSK3β, and GYS play vital roles in glycogen synthesis [47]. Our study indicated that PGLP up-regulated the mRNA expression levels of *pi3k*, *akt*, and *gys* and down-regulated the mRNA expression levels of *gsk3β* of muscle in grass carp. However, the effect of PGLP on grass carp muscle glycogen synthesis has not been reported, but this is similar to the study of other plant extracts. For instance, dendrobium officinale (*Dendrobium officinale* Kimura & Migo) polysaccharides increased glycogen content and the protein expressions of PI3K, Akt, GSK3β, and GYS in muscle of mice [48]. Glucose is an important substrate for glycogen synthesis, so the increase of glycogen synthesis is closely associated with the increase of glucose content in muscle [49]. Meanwhile, GLUT4 is vital in regulating glucose uptake by muscle cells [50]. In this experiment, PGLP enhanced the expression level of *glut4* mRNA in muscle, indicating that more glucose was transported into muscle cells to participate in glycogen synthesis. To sum up, the above results suggested that PGLP may promote energy metabolism by promoting the phosphate system and aerobic glycolysis and reducing anaerobic glycolysis. At the same time, PGLP promoting energy metabolism may be related to promoting the conversion of pyruvate to acetyl-CoA into the TCA cycle, thereby enhancing OXPHOS. Therefore, we next studied OXPHOS, the main way of ATP production.

### PGLP may promote oxidative phosphorylation by maintaining mitochondrial homeostasis

Mitochondria are vital sites of the OXPHOS process. Mitochondrial complexes play an important role in OXPHOS [51]. In this experiment, PGLP increased the protein expressions of NDUFV1, SDHB, UQCRC2, COX IV, and ATP5A1 in muscle, demonstrating that it enhanced OXPHOS. The function of mitochondria is directly associated with the quantity, quality, and morphological structure of mitochondria. The quantity and quality of mitochondria are regulated by biogenesis and autophagy [52]. Mitochondrial morphology is regulated by fission and fusion. In our study, PGLP enhanced the protein expressions of LKB1, AMPK, p-AMPK, PGC-1α, and Nrf1 and down-regulated the mRNA expression levels of *pink1*, *parkin*, *bnip3*, *nix1*, *nix2*, *drp-1*, and *fis1*, decreased the protein expressions of P62 and LC3, and up-regulated *mfn1/2* and *opa-1* mRNA expressions of muscle in grass carp. It was demonstrated that PGLP promoted mitochondrial biogenesis and fusion, as well as inhibited autophagy and fission to maintain mitochondrial homeostasis. Until now, the effect of PGLP on animal mitochondrial quality control has not been reported. Mitochondria with low membrane potential generated by mitochondrial division either restore the membrane potential to fuse with other mitochondria or maintain the depolarization state to be cleared by autophagy [53]. Meanwhile, studies found that PGRP increased the mitochondrial membrane potential of cardiomyocytes after hypoxia/reoxygenation injury [54]. Therefore, we speculated that PGLP may enhance the recovery of low mitochondrial membrane potential, promote mitochondrial fusion and inhibit autophagy, but further verification is needed.

## Conclusions

Our study found that PGLP improved growth performance, muscle nutritional value, texture characteristics, and alleviated the rate of pH decline, thereby improving flesh quality in fish. The improvement of flesh quality may be related to the promotion of growth and development of myofibers, which is proved by the promotion of PGLP on hyperplasia of myofibers in grass carp. Meanwhile, energy metabolism is closely related to flesh quality. We found that PGLP improved the aerobic glycolysis, and OXPHOS in muscle via increasing the contents of creatine, glycogen, and acetyl-CoA and the activity of CK, PK, and HK. Besides, PGLP increased mitochondrial function via maintaining the dynamic equilibrium among mitochondrial biogenesis, autophagy, fission, and fusion in muscle. Finally, according to the growth performance, the recommended amount of PGLP in fish feed is 100–200 mg/kg.

## Supplementary Information

Additional file 1: Table S1 The components and nutritional makeup of the basal diet. Table S2 The calculation formula of grass carp growth performance. Table S3 The biochemical index determination kit number. Table S4 The real-time PCR primer sequences.

## Data Availability

Data will be made available on request.

## Abbreviations

COX IV: Cytochrome c oxidase
DRP-1: Dynamin-related protein-1
FIS1: Fission protein 1
GLUT4: Glucose transporter-4
GYS: Glycogen synthase
GSK3β: Glycogen synthase kinase-3β
HK: Hexokinase
LC3: Microtubule-associated protein 1 light chain 3
LDH: Lactate dehydrogenase
LKB1: Liver kinase B1
MFN1/2: Mitofusin1/2
MRFs: Myogenic regulatory factors
NDUFV1: NADH-ubiquinone oxidoreductase core subunit V1
Nrf1: Nuclear respiratory factor 1
OPA-1: Optic atrophy-1
OXPHOS: Oxidative phosphorylation
P62: Autophagy receptor P62
Parkin: E3 ubiquitin-protein ligase parkin
PFK: Phosphofructokinase
PGC-1α: Proliferator-activated receptor gamma coactivator-1α
Pink1: PTEN-induced putative kinase 1
PI3K: Phosphoinositide 3-kinase
PK: Pyruvate kinase
PGLP: *Panax ginseng* leaf polysaccharide
PGRP: *Panax ginseng* root polysaccharide
SDHB: Succinate dehydrogenase B
UQCRC2: Ubiquinol-cytochrome c reductase core protein 2
