# Supplementary material for: A new vision of Panax ginseng leaf polysaccharide function: multiple roles in improving growth, flesh quality and muscle energy metabolism of sub-adult grass carp (Ctenopharyngodon idella)
Source: J Anim Sci Biotechnol. 2025 Nov 14;16:149. doi: 10.1186/s40104-025-01256-z (PMC12616924; doi:10.1186/s40104-025-01256-z)
Supplement: Supplementary file 1 — Additional file 1: Table S1 The components and nutritional makeup of the basal diet. Table S2 The calculation formula of grass carp growth performance. Table S3 The biochemical index determination kit number. Table S4 The real-time PCR primer sequences. [file 40104_2025_1256_MOESM1_ESM.docx]

**Additional file 1**

**Table S1** Formulation and nutrients composition of experimental diets

| **Ingredients** | **Content, g/kg** | **Nutrients content^5^** | **%** |
| --- | --- | --- | --- |
| Fish meal | 30.00 | Crude protein | 26.14 |
| soybean meal | 200.00 | Crude lipid | 4.82 |
| cottonseed meal | 190.00 | Available phosphorus | 0.40 |
| Rapeseed meal | 142.80 | n3:n6 | 1.03 |
| Fish oil | 24.60 | n3 + n6 | 1.96 |
| Soybean oil | 9.40 |  |  |
| Wheat flour | 68.05 |  |  |
| α-starch | 250.00 |  |  |
| Ca(H_2_PO_4_)_2_ | 12.40 |  |  |
| Microcrystalline cellulose | 20.00 |  |  |
| Vitamin premix^1^ | 10.00 |  |  |
| Mineral premix^2^ | 20.00 |  |  |
| PGLP premix^3^ | 10.00 |  |  |
| Choline chloride premix^4^ | 10.00 |  |  |
| Butylated hydroxyanisole (99%) | 0.15 |  |  |
| Thr (97.5%) | 1.60 |  |  |
| Trp (98%) | 1.00 |  |  |
| Total | 1000.00 |  |  |

^1^ Per kilogram of Vitamin premix (g/kg): retinyl acetate (5000,000 IU/g), 0.44 g; cholecalciferol (500,000 IU/g), 0.19 g; tocopherol acetate (50%), 25.50 g; menadione (50%), 0.38 g; cyanocobalamin (1%), 0.94 g; D-biotin (2%), 1.05 g; folic acid (95%), 0.17 g; thiamine nitrate (98%), 0.11 g; ascorbic acid (95%), 9.77 g; niacin (99%), 3.44g; inositol (96.5%), 28.67 g; calcium-D-pantothenate (93.1%), 4.42 g; riboflavin (80%), 0.73 g; pyridoxine hydrochloride (98%), 0.55 g. All ingredients were diluted with corn starch to 1 kg

^2^ Per kilogram of mineral premix (g/kg): MnSO_4_ H_2_O (31.8% Mn), 2.66 g; MgSO_4_ H_2_O (15.0% Mg), 256.79 g; FeSO_4_ H_2_O (30.0% Fe), 12.61 g; ZnSO_4_ H_2_O (34.5% Zn), 8.87 g; CuSO_4_ 5H_2_O (25.1% Cu), 0.95 g; Ca (IO_3_)_2_ (3.2% I), 1.56 g; yeast selenium (0.2% Se), 13.65 g; and all ingredients were diluted with corn starch to 1 kg

^3^ Per kilogram of PGLP premix (g/kg): the corresponding ratio of PGLP, and filled with microcrystalline cellulose to 1 kg

^4^ Per kilogram of choline chloride (g/kg): choline chloride (50%), 261.95 g, the rest was diluted with corn starch to 1 kg

^5^ The contents of crude protein and crude fat in feed were measured. The contents of n3, n6 and available phosphorus were calculated

**Table S2** The calculation formula of related indicators

| **Index** | **Formula** |
| --- | --- |
| Weight gain (WG) | WG (g/fish) = final weight (g/fish)-initial weight (g/fish) |
| Weight gain percentage (PWG) | PWG (%) = (final weight-initial weight) (g/fish)/ initial weight (g/fish) × 100 |
| Specific growth rate (SGR) | SGR (%/d) = (lnWt-lnW_0_) × 100/t |
| Feed conversion ratio (FCR) | FCR = (feed intake in dry matter, g)/[(final body weight, g) − (initial body weight, g)] |
| Feed efficiency (FE) | FE = [final body weight (g/fish) − Initial body weight (g/fish)]/FI (g/fish) |
| Feed intake (FI) | FI (g/fish) = total feed consumption (dry) (g/fish) − total uneaten feed (dry) (g/fish) |
| Condition factor (CF) | CF (g/cm^3^) = [final body weight (g/fish)/body length (cm)^3^] × 100 |
| Flesh rate | Flesh rate (%) = [carcass weight (g/fish) – fishbone weight (g/fish)]/FBW (g/fish) × 100 |
| Cooking loss | Cooking loss (%)= (W_1_-W_2_)/W_1_ × 100 |

Note: W_t_ represents the final weight of the test fish, unit g/fish; W_0_ represents the initial weight of the test fish, unit g/fish; t represents the number of experimental days, here is 60 d; W_1_ represents the sample weight before water bath treatment (70℃), unit g; W_2_ represents the sample weight after water bath treatment (70℃), unit g

**Table S3** Biochemical index determination kit number

| **Index** | **Assay method** | **Kit number** |
| --- | --- | --- |
| Adenosine triphosphate content (ATP) | colorimetric method | A095-1-1 |
| Lactic acid | colorimetric method | A019-2-1 |
| Muscle glycogen | colorimetric method | A043-1-1 |
| Pyruvate | colorimetric method | A081-1-1 |
| Creatine kinase activity (CK) | colorimetric method | A032-1-1 |
| Pyruvate kinase activity (PK) | colorimetric method | A076-1-1 |
| Phosphofructokinase (PFK) | colorimetric method | A129-1-1 |
| Hexokinase activity (HK) | colorimetric method | A077-3-1 |
| Lactate dehydrogenase (LDH) | micro-plate method | A020-2-2 |
| Creatine | micro-plate method | BL889B |
| Acetyl-CoA | micro-plate method | YJ593299b |

Note: the creatine and acetyl-CoA content determination kits were purchased, respectively, from biosharp of Hefei Labgic Technology Co.Ltd (Anhui, China) and Shanghai Enzyme-linked Biotechnology Co., Ltd. (Shanghai, China); the remaining kits were purchased in Nanjing Jiancheng Bioengineering Institute (Nanjing, China)

**Table S4** Real-time PCR primer sequences

| **Target gene** | **Primer sequence forward (5'-3')** | **Primer sequence reverse (5'-3')** | **Accession number** |
| --- | --- | --- | --- |
| *myod* | CCCTTGCTTCAACACCAACG | TCTCCTCTCCCTCATGGTGG | GU218462 |
| *myf5* | GTGCCTGTGCCTCATCTCCT | AATGCGTGGTTCACCTTCTTCA | GU290227 |
| *mrf4* | GAAAATCTGCTCCAACTACC | CGCTGCGTAAAATCTCCA | KT899334 |
| *myog* | AGAGGAGGTTGAAGAAGGTC | GTTCCTGCTGGTTGAGAGA | JQ793897 |
| *myhc* | GCTCATCACCACCAACCC | GCCTCCTCTGTGCCATCA | AB255030 |
| *mstn* | GCAGGAGTCACGTCTTGGCA | GAGTCCCTCCGGATTCGCTT | KM874826 |
| *pi3k* | AGTCAGTGCCTGTGGCTGAG | CGTGTCCATGACCTCAGAGC | KY763989 |
| *akt* | CCTGGTGATGAAGGAGCTGA | CTGTCAGAGAGCCTCCAGCA | KY763985 |
| *gsk3β* | TCGGCTGTTGGAGTACACAC | TAGAGGCGTTGGTGGGAGTA | MK770659 |
| *gys* | CCTCCAGTAACAACTCACAACA | CAGATAGATTGGTGGTTACGC | JQ792167 |
| *glut4* | TCTATTGGGGGCATGGTGTC | GAGTTGAAGGTGGTCTCAT | [1] |
| *pink1* | TGCGGAGACTGAGAAGTG | CCATAGACATAGAGCGAAGGAT | MZ358119 |
| *parkin* | TGGACTTGGCTGTGGGTTT | CACGGGAACTTGGCATTTGG | MZ358120 |
| *bnip3* | GCGTTGCTCTTGGTAGATTGC | GGGAGTGGAAAGTTCTGTGGG | MW032445.1 |
| *nix1* | CCAGTCGGCCAGAGAACATT | AGGAACTCAGCGGAGAACAC | MW032446 |
| *nix2* | GGGAGCCTGGATTAAACGGG | CCGACCATCCCATCATCCTC | MW032447 |
| *drp1* | GGATGGAGAAGAGTGGGGGA | TGGGACCTTTGTGATGCCAG | CI_GC_1332 |
| *mfn-1* | GTTTGGATGCTGGCTGTCT | GCTTTGAGAGGCGTTCGTTG | CI_GC_23762 |
| *mfn-2* | AAAGTGGCAGGGATTGGGG | CGTAGGAAGCAGTTGGTGGT | CI_GC_11929 |
| *opa-1* | TCCATCACCGCTACTTTGCT | GGGATTTGGGAGGACGCAT | CI_GC_27017 |
| *fis-1* | CAGACATAGTAGCACCAGAA | TCCAATACCACCGACAATC | [2] |
| *β-actin* | GGCTGTGCTGTCCCTGTA | GGGCATAACCCTCGTAGAT | M25013 |

**References**

[1] Li HD, Song L, Wei MK, Yuan XT, Wu WY, Kaneko G, et al. DHA promotes healthy adipose tissue remodeling and carbohydrate utilization in grass carp (*Ctenopharyngodon idellus*) and largemouth bass (*Micropterus salmoides*). Aquacult Rep. 2023;33:101856-67. <http://dx.doi.org/10.1016/j.aqrep.2023.101856>

[2] Shi B, Liu Q, Xu C, Zhang Z, Cai J. Chlorantraniliprole induces mitophagy, ferroptosis, and cytokine homeostasis imbalance in grass carp (*Ctenopharyngodon idella*) hepatocytes via the mtROS-mitochondrial fission/fusion axis. Pestic Biochem Physiol. 2024;200:105830-42. <http://dx.doi.org/10.1016/j.pestbp.2024.105830>
